# Waterborne Intumescent Coatings Containing Industrial and Bio-Fillers for Fire Protection of Timber Materials

**DOI:** 10.3390/polym12040757

**Published:** 2020-03-31

**Authors:** Abderrahman Aqlibous, Svetlana Tretsiakova-McNally, Talal Fateh

**Affiliations:** 1 INSA Centre Val de Loire, 88 boulevard Lahitolle, CS60013, 18022 Bourges CEDEX, France; aqlibous.a@gmx.com; 2 Belfast School of Architecture and the Built Environment, Ulster University, Newtownabbey BT37 0QB, Northern Ireland, UK; 3 EFECTIS UK& Ireland, Newtownabbey BT37 0QB, UK; talal.fateh@EFECTIS.COM

**Keywords:** cone calorimeter, intumescent coatings, bio-fillers, wood, flame retardants

## Abstract

Flammability and combustion of softwood treated with intumescent coatings were studied in the present work. The formulations applied onto wood surfaces contained different ratios of industrial fillers, titanium dioxide TiO_2_ and aluminium trihydroxide Al(OH)_3_, and/or bio-fillers, eggshell and rice husk ash. Combustion behaviours of unprotected and protected wood samples have been examined with the aid of cone calorimetry performed under the varied levels of thermal flux ranging from 30 to 50 kW/m². The char residues obtained after the completion of cone calorimetry test at 40 kW/m² were analysed by the Raman spectroscopy. The fire protective properties of the studied coatings were strongly influenced by the nature of the fillers as well as by the intensity of thermal irradiance. The incorporation of bio-based fillers into the water-based intumescent formulations significantly improved fire resistance of wood substrates. For example, at 30 kW/m², the Effective Heat of Combustion was reduced by more than 40%, whilst the average Peak to Heat Release Rate had dropped from 193.2 to 150.3 kW/m² for the wood sample protected with the formulation incorporating two industrial and two bio-fillers. Moreover, an application of the studied coatings resulted in a notable reduction of the back surface temperature of the wood specimens.

## 1. Introduction

As one of the oldest traditional construction materials, timber is extensively used in modern construction projects, for example for manufacturing beams, joists, columns, cords, roofs, walls, floors, etc. It comes in different forms such as plywood, panels or boards. Although timber is not as strong as concrete and steel, it is significantly lighter, which makes it a good choice for long-span and tall structures. Its light weight is also a great advantage for seismic resilient structures due to better resistance to the impacts of earth shaking [1]. Another benefit of timber is in its sustainability and renewable nature. Frequently, it is not possible to utilize raw wood for real-life applications and, thus, it should be protected from detrimental impacts of moisture, fungi, bacteria, insects, UV light and fires [2]. There are three complementary strategies to achieve the required level of timber protection: changes to the wood cell walls through thermal or chemical modifications, impregnation and coatings.

In the 20th century, the use of wood-based materials was restricted by prescriptive regulations mainly due to their relatively high flammability levels [3]. It is worth mentioning that wood is a largely heterogeneous composite material, the computer modelling of which is a challenging task [1]. However, owing to the constantly improving simulation modelling techniques for dimensioning and also with the integration of the performance-based design in the EU common standards [4], the use of timber for large and tall structures has been on the increase in recent years. This, combined with the growing concerns about the environment protection, make wood an attractive material for the future constructions.

Depending on its original design and the degradation level, a wood-based product in the end of its life can be either re-used (intact or re-sized), or serve as a source for the new value-added products. The unwanted wooden parts can also be burnt to generate energy, thus resulting in an almost carbon neutral process. However, the last solution can be harmful to the environment, especially if timber was treated with non-eco-friendly, toxic fire retardants, additives, coatings or adhesives. In this case, the burning of the treated wood must be carried out at the appropriate combustion facilities with a special equipment to reduce the amounts of air and water pollutants generated [1].

The pyrolysis and combustion of timber is well studied, and the flammability pattern is described in detail [5]. According to the Eurocode [4], timber can lose almost 50% of its strength when heated to temperatures of 100 °C and higher. On the other hand, the thermal conductivity of wood is lower compared to other construction materials (e.g., steel and concrete), and when its surface is carbonized, the formed char layer acts as an incombustible thermal insulation. There are several means to protect the wood from the impact of fires including impregnation, plasma treatments and surface coatings [5,6]. Among many options currently available, our attention was drawn to the use of intumescent formulations as a route to improve fire performance of timber. This is a very efficient and versatile method of fire protecting different materials (metal, plastic, textiles, timber, etc.), both for indoor and outdoor uses. The intumescent coatings can be easily applied onto the new elements during construction or on the existing structures using gun sprayers, brushes and rollers. Another notable advantage of intumescent formulations is that once applied they do not alter the intrinsic properties of the protected substrate.

Generally, intumescent coatings are composed of an acid source, a carbon source, and a blowing agent, with the addition of a range of binders, pigments and additives that improve different characteristics (e.g., processing, adhesion, cohesion, etc.) [7]. The main components of intumescent coatings, also used in the current work, include: ammonium polyphosphate (APP) as an acid source, melamine as a blowing agent, and pentaerythritol (PER) as a source of carbon. Alongi et al. [8] reported the most common examples of traditional components for the intumescent formulations. In the study carried out by C. de Sáa et al. [9] it was highlighted that the main raw materials for the manufacturing of the intumescent coatings are still largely rely on non-renewable sources, the processing of which are frequently associated with the negative impacts on the environment. Moreover, the most effective formulations to fire proof wood-based materials contain halogens, and have a tendency to generate toxic and irritant gaseous products in the fire effluent. Thus, it is essential to find the suitable alternatives to these components by incorporating, for example, natural and environmentally friendly materials, industrial by-products, agricultural and food waste. Nowadays, different research groups are working on the development of halogen-free intumescent coatings containing bio-fillers. The review carried out by Costes et al. [10] discussed a wide diversity of bio-based flame retardants that currently exist. For example, C. de Sá et al. [9] investigated the efficiency of ginger and coffee husk as a carbon source along with zinc phosphate and tripolyphosphate as non-halogenated flame retardants. The intumescent coatings with micro- and nano-eggshell were also studied by Yew et al. [11,12], who showed that the smaller eggshell particles provide higher homogeneity and better thermal insulation. The mollusk shells were studied for their thermal insulation properties by Zhang et al. [13], Sophia and Sakthieswaran [14]. The synergistic effects of natural-based tea saponin in intumescent flame-retardant coatings have been investigated in detail by Qian et al. [15]. An addition of an organically modified montmorillonite (MMT) to a styrene-acrylic emulsion, with the following application onto a plywood, greatly improved its fire resistance [16]. Hu et al. [17] demonstrated that CaAlCO_3_-layered double hydroxides (LDHs) incorporated into an intumescent formulation for steel structures improved its ability to dilute a flammable gas, and catalysed the reaction between APP and PER. Guo et al. [18] reported the enhanced flame retardancy and smoke suppression attributes of Mg-Al LDH-based coatings for wood substrates. In the most recent publication by Hu et al. [19] the synergistic action of MMT within water-based intumescent coating was proved to be effective in relation to fire resistance of plywood. All these studies demonstrate that bio-fillers and non-halogenated flame retardants could increase the thermal insulation of the protected substrates while decreasing the toxic potencies of gaseous products of combustion. Since the bio-fillers often come from food waste (e.g., eggshells, seashells, and rice husk), their use also presents environmental and economic benefits.

Nasir et al. [20] studied the effect of intumescent coatings with titanium dioxide, aluminium trihydroxide, rice husk ash and eggshell in fire-proofing steel surfaces. In the current study, the formulations tested by Nasir et al. [20] were applied onto the wood surfaces and their combustion characteristics were determined with the aid of a cone calorimeter.

## 2. Materials and Methods

### 2.1. Substrate

The wood substrate used in the present work was knotless *Radiata* Pine (ML Panel, UK). Prior to the study the wooden boards were stored in a conditioning room at 23 ± 2 °C and with 50 ± 5% humidity for at least one week according to the ISO 554:1976 [21]. The square pieces with the size 100 × 100 × 20 mm were cut as per requirements of the ISO 5660-1:2015 [22]. The moisture content was evaluated on six wood samples taken at random and in accordance with BS EN 14774-2:2009 [23]. The average moisture content of wooden substrate was found to be 10.21 wt.%.

### 2.2. Intumescent Coatings

The six waterborne coatings, labelled as A–F (Table 1), were produced at the University of Malaya. The main components of the intumescent formulations included: vinyl acetate copolymer (VAC) (29.15 wt.%) as a binder; ammonium polyphosphate (APP) (18.50 wt.%) as an acid source; pentaerythritol (PER) (9.25 wt.%) as a carbon source; melamine (9.25 wt.%) as a blowing agent; and water (23.85 wt.%). The varied amounts of industrial fillers, i.e., titanium dioxide (TiO_2_) and aluminium trihydroxide (Al(OH)_3_), and biological fillers, i.e., Rice Husk Ash (RHA) and Eggshell (ES) were incorporated into these formulations in the quantities shown in Table 1. The binder VAC, the acid and the carbon sources (APP and PER) were supplied by International Chemical Ltd. (China). The industrial fillers were bought from the Scientific Group Sdn. Bhd. (Malaysia). The biological fillers came from a rice milling factory and a chicken farm (Malaysia). TiO_2_ as a non-combustible pigment has a high efficiency, thus making it indispensable in all the formulations studied. Al(OH)_3_ is used as a flame retardant and a char-forming agent. RHA has a high silica (SiO_2_) content, while ES contains up to 95% of calcium carbonate (CaCO_3_)_._ SiO_2_ and CaCO_3_ also improve the mechanical and thermal properties of the coatings. The proportions and roles of the constituents in each coating have been discussed earlier by Nasir et al. [20].

### 2.3. Application of Intumescent Coatings

**Figure 1:**
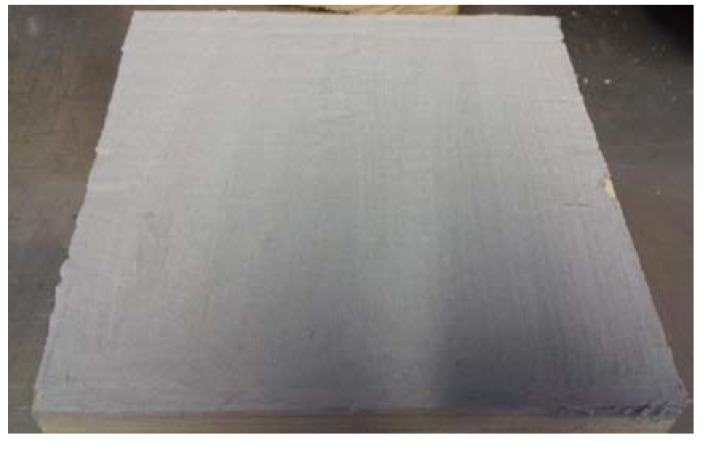
A coated sample of wood.

The A–F coatings (Table 1) were applied, at room temperature, onto a surface of wood substrates as per following protocol:

1. The initial mass of the substrate was measured before applying the coating.
2. The sides of the wood sample were covered with a tape to ensure that only one square surface was treated.
3. A first layer of each formulation was applied with a paintbrush.
4. The tape was then removed to get rid of the excess paint accidentally leaked on to the sides.
5. The painted substrate’s weight was measured. The mass difference provided the amount of formulation applied for each sample.
6. Some extra coating was applied, if necessary, to reach the desired mass. The final coated samples (Figure 1) were then placed in a fume hood (at the air flux of 0.030 m^3^/s) for 1 h before being transferred to the conditioning room for a minimum of one week.

The wood samples, prepared in duplicates, were covered uniformly with the mean area density of 505.17 g/m², thus giving the mean thickness of the applied coating of around 0.4 mm. The following naming convention was used in the study: the unprotected wood sample was labelled as “W”, the wood sample treated with the formulation A was labelled as “WA”, the wood sample treated with the formulation B was labelled as “WB”, etc.

### 2.4. Cone Calorimeter

The combustibility of the untreated and treated wood samples has been evaluated using a cone calorimeter in conformance with the ISO 5660-1:2015 [22]. The apparatus and the technique are extensively detailed by Babrauskas [24]. The samples were tested under three different heat fluxes: 30, 40 and 50 kW/m². The air flow rate of the fume hood was set at 0.0240 ± 2 m^3^/s at the beginning of the test. The ignition was triggered by a 10 kV spark igniter. The temperature of the back surface of the samples was measured by a K-type thermocouple. The sample holder was the same as the one employed by Nasir et al. [20]. The bottom surface of the cone heater and the top of the tested samples were separated by a 25 mm air gap according to the ISO 5660-1:2015 [22].

### 2.5. Raman Spectroscopy

The char residues formed on the surface wood after the cone calorimeter test were collected and ground to a fine powder using a pestle and a mortar. The Raman spectra were recorded on the char samples using a confocal Raman microscope system (inVia Qontor; Renishaw Inc., UK), with a 532 nm laser and equipped with a Nikon objective lens (50×). The spectra were collected over three sweeps of 10 s each and a 50% filter, with a spectral resolution of 1 cm^−1^.

## 3. Results and Discussion

### 3.1. Cone Calorimeter Results

#### 3.1.1. Heat Release Rates and Peaks to Heat Release Rate

The Heat Release Rate (HRR) quantitatively characterizes the power of a fire. It is considered to be “the most important variable of a fire hazard” [25]. This is the power (kW), released by a material’s surface (m^2^) during the degradation under the thermal stress. The higher this value for a material or a product, the more it will contribute to the destructive effects of a fire (e.g., embrittlement and destruction of structures, propagation of flames, physical impact on people, etc.). The HRR value varies over time during the development of the fire, which is evaluated from the levels of oxygen O_2_ consumption [26].

**Figure 2:**
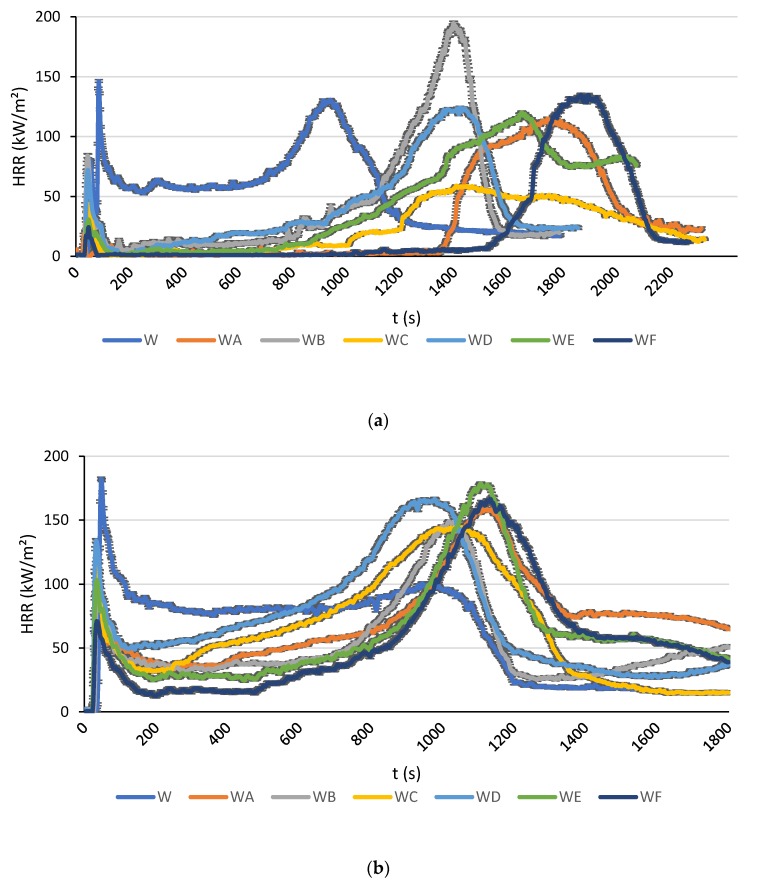

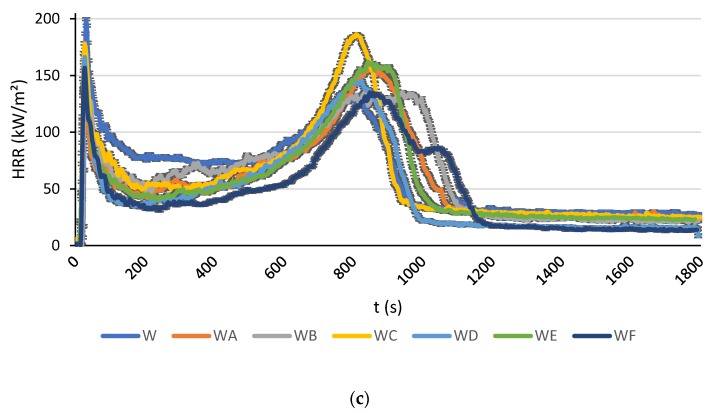
The Heat Release Rate (HRR) as a function of time for a thermal flux of: (**a**) 30 kW/m²; (**b**) 40 kW/m²; (**c**) 50 kW/m².

**Figure 3:**
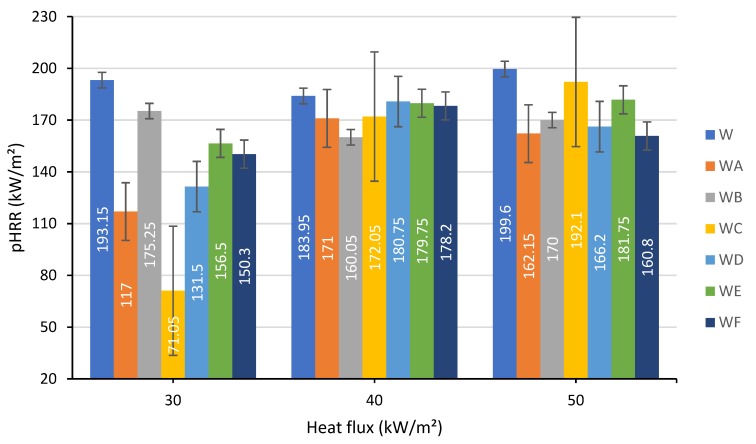
The average peaks of HRR measured at 30, 40 and 50 kW/m² of heat flux.

The time dependencies of the average HRR values and the peaks of Heat Release Rate (pHRR) for the tests at 30, 40 and 50 kW/m² are shown in Figure 2 and in Figure 3.

As it follows from Figure 2a, at the heat flux of 30 kW/m², all the tested coatings attenuate the HRR compared to the unprotected wood. They also limit the first peak that corresponds to the ignition, which is common for the mechanism of intumescence. After a few moments, the protective layer reacted to the heat exposure and inhibited the flaming combustion, thus decreasing the heat produced and limiting the production of the pyrolysis gases. Obviously, after a certain time all the curves of the protected specimens exceeded the one of the unprotected specimens because the protective effect on the coating was temporary. The unprotected wood burnt directly and when most of the fuel was consumed the HRR began to fall. In the case of the coated specimen, the protective layer degraded progressively until the moment when the heat flux from the cone directly attacked the wood substrate, resulting in some increase of the HRR during the last stages of testing. It is observed that the coatings A (TiO_2_ + RHA) and F (all four fillers) were capable of containing the rise of HRR for longer periods of time. It is also worth noting an excellent performance of the coating C, with incorporated TiO_2_ and ES bio-filler, which kept the HRR relatively low for longer times. Indeed, after the initial peak corresponding to ignition, the HRR value remained below 60 kW/m², reduced by almost a half compared to the unprotected wood (Figure 2a).

At 40 kW/m², the HRR curves of the coated specimens began to converge towards the one of the unprotected materials. It was difficult to determine which coating offered the best result regarding the reduction of the HRR. As it follows from Figure 2b, from the start of the test to about 975 s, the HRR for the sample with the coating F was lower than that of the other protected specimens, but afterwards it became somewhat higher compared to other samples over the remaining time interval.

At 50 kW/m², all the profiles of HRR vs. time looked very similar but with some differences in the average HRR values. It has been observed during the tests (and confirmed by these results) that the intumescent coatings did not have sufficient time to react when exposed to the higher heat flux levels. The high intensity of thermal radiation destroyed the paint layer before the chemical reactions started to operate. The large cracks appeared very quickly on the surface of the coatings, allowing pyrolysis gases to escape from the combustible substrate. These gases fed the flames on the surface, increasing the radiation received by the intumescent layer, inevitably accelerating its destruction.

Similar overall trends were observed for the values of the peak Heat Release Rate (pHRR) (Figure 3). The pHRRs were distinct from each other at a lower heat flux of 30 kW/m², while the differences were narrowed at higher levels. Nevertheless, regardless of the level of heat flux, the application of all tested coatings had led to a reduction in the pHRR values. At 30 kW/m², the coating C containing TiO_2_ and ES, performed better than others: pHRR was reduced to 71.05 kW/m² compared to 193.15 kW/m² for the unprotected wood. On the other hand, at 50 kW/m², the coating C was rated as the worst performing in regard to reducing the pHRR; it was merely better than the original wood sample. At this level of heat exposure, the application of F, A, D and B coatings onto wood surface lowered the pHRR from 199.6 kW/m^2^ for the unprotected wood to: 160.8 kW/m² for the wood painted with the formulation F; 162.15 kW/m² for one with the formulation A; 166.2 kW/m² for one with the formulation D; and 170.0 kW/m² for one with the formulation B. Thus, we can observe that the intumescent formulation that combines both industrial fillers (TiO_2_ and Al(OH)_3_) and bio-fillers (RHA and ES) was the most efficient in terms of controlling the rates of heat release at higher heat flux levels. Under the influence of the radiative heat, the intumescent coatings expand, thus leading to a drop in the quantity of heat received by the wood surface. The obtained results correlate with the findings of the earlier study involving the application of similar formulations on to steel substrates [20]. However, any statement related to the HRR is limited due to the high uncertainty linked to the measurements.

#### 3.1.2. Effective Heat of Combustion

The Effective Heat of Combustion (EHC), expressed in MJ/kg, is an important parameter for the characterization of a material as it indicates the energy released per unit of mass burnt.

This parameter is calculated from the Equation (1).

(1) $$EHC=\frac{\int_{t_{0}}^{t_{f}}HRR\left(t\right)dt}{m_{lost}},$$

withEHC:Effective Heat of Combustion(kJ/g or MJ/kg)
$t_{0}$:Time of test beginning(s)
$t_{f}$:Time of test end(s)
HRR(t):Instantaneous Heat Release Rate(kW)
t:Time(s)
$m_{lost}$:Total mass lost from t_0_ to t_f_(g)

**Figure 4:**
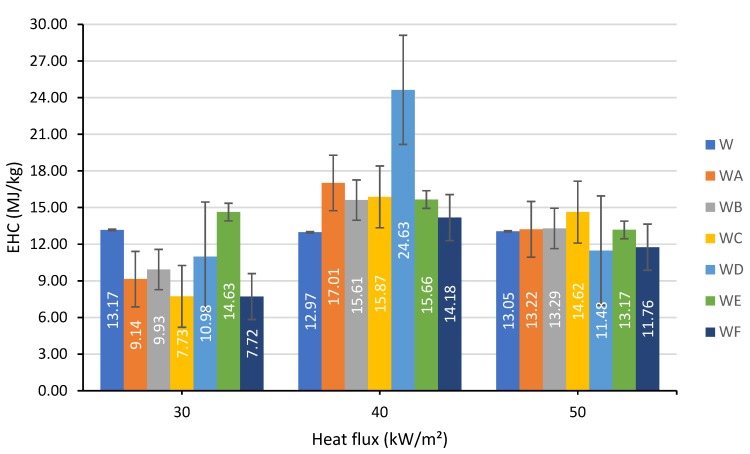
The values of Effective Heat of Combustion (EHC) determined at 30, 40 and 50 kW/m² of heat flux.

The average EHC values for tests carried out at different heat fluxes, 30, 40 and 50 kW/m², are shown in Figure 4.

The raw wood had an average EHC of about 13 MJ/kg regardless of the heat flux applied. It was observed that at 30 kW/m², the EHC of unprotected wood was generally higher than that of the painted wood samples, with the exception of the formulation E. The pattern was completely opposite at 40 kW/m² when the uncoated wood sample had the lowest EHC, whilst the sample coated with formulation D, incorporating two industrial fillers and RHA, was characterized by the highest EHC value, twice as high compared to the unmodified wood. In contrast, when the heat flux was increased further to 50 kW/m² the formulation D had reduced the EHC to the lowest value of 11.48 MJ/kg. The coating that demonstrated a consistent reduction of the EHC for the studied fluxes was based on the formulation F containing all four fillers (see Table 1).

It is interesting to note that at heat flux of 40 kW/m² all painted specimens had the EHC values higher than those measured at 50 kW/m². It can be assumed that the growth of the radiant heat, from 40 to 50 kW/m², is associated with overcoming a certain temperature threshold that is required for triggering endothermic chemical reactions. The chemistry behind these chemical processes is presented in the Equations (2)–(6) and discussed by Nasir et al. [20]. The fire-retardant effect of the formulations is largely provided through the endothermic decomposition reactions. For example, aluminium trihydroxide dehydrates, liberating water vapour and aluminium trioxide as per Equation (3). The endothermicity of the process (3) reduces the overall temperature, the water vapours thus formed cool the wood substrate and dilute flammable gases. Additionally, the produced aluminium trioxide contributes to a formation of the protective layer and also promotes char oxidation. The production of water vapours is enhanced in the formulation containing RHA enriched with silica (reactions (4) and (5)), while the incorporation of ES, containing calcium carbonate, boosts the production of carbon dioxide acting as a diluent and a blowing agent, as per Equation (6).

(2) $$TiO_{2}\to Ti+O_{2},$$

(3) $$2Al{\left(OH\right)}_{3}\to Al_{2}O_{3}+3H_{2}O,$$

(4) $$2NaOH+SiO_{2}\to Na_{2}SiO_{3}+H_{2}O,$$

(5) $$Na_{2}SiO_{3}+H_{2}SO_{4}\to SiO_{2}+Na_{2}SO_{4}+H_{2}O,$$

(6) $$CaCO_{3}\to CaO+CO_{2},$$

#### 3.1.3. Time to Ignition

**Figure 5:**
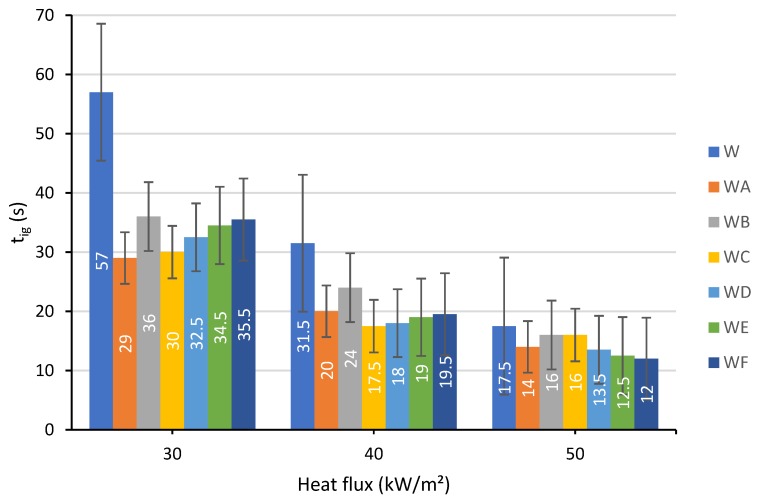
Time to ignition determined at a heat flux of: 30, 40 and 50 kW/m².

The time to ignition (t_ig_) values of unprotected and projected wood samples measured at 30, 40 and 50 kW/m² are presented in Figure 5**.**

The first, rather direct and logical observation that can be made from the data shown in Figure 5 is that the time to ignition decreases as the flux increases in all cases. On the other hand, it seems a bit counterintuitive, but all the painted specimens ignite faster than the unprotected counterparts, regardless of the flux intensity. However, the differences, especially at the higher thermal radiation levels, are not significant enough to provide a reliable ranking of the tested coatings. At a lower heat flux level (30 kW/m^2^), the flames originating from the coating accelerate the charring processes. When the heat flux is intensified, the impact of the coating on the ignition times is limited as the wood was burnt very fast, while the coating protection layer may require more time to start an effective thermal shielding. The incorporation of only industrial hydrated fillers into the intumescent formulation (coating B) led to slightly higher values of time to ignition, which is due to the dilution of combustible volatiles in the gaseous phase [20]. In addition, it was found that an application of the coating C, with TiO_2_ and ES, increased the total burning time of softwood by 532 s.

#### 3.1.4. The Flaming Duration

**Figure 6:**
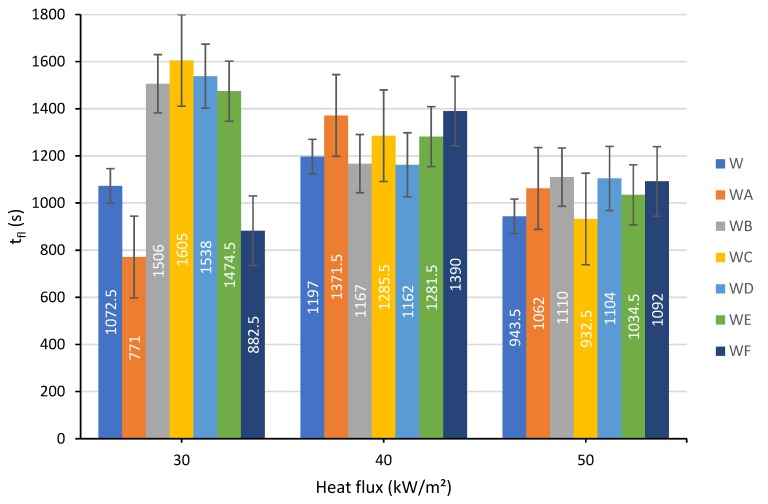
The flaming duration measured at heat flux of: 30, 40 and 50 kW/m².

The flaming durations measured at 30, 40 and 50 kW/m² of heat flux for all the samples tested in the study are presented in Figure 6.

As it follows from Figure 6, at the heat flux of 30 kW/m², the application of the coating A, containing titanium dioxide and rice husk ash, and the coating F, combining all four fillers, significantly shortened the flaming duration compared to the uncoated wood substrate; whereas the formulations B, C, D and E prolonged the flaming stage. At higher levels of thermal flux, 40 and 50 kW/m², it was not possible to identify the best coating option due to the lack of a clear trend. However, the duration of the flame can be investigated alone without measuring other parameters such as HRR.

#### 3.1.5. Production of Carbon Monoxide and Carbon Dioxide

**Figure 7:**
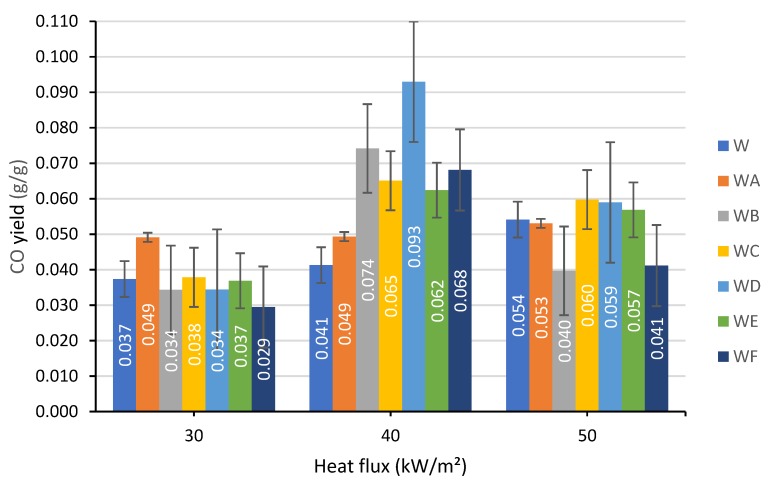
The CO yields measured at a heat flux of: 30, 40 and 50 kW/m².

**Figure 8:**
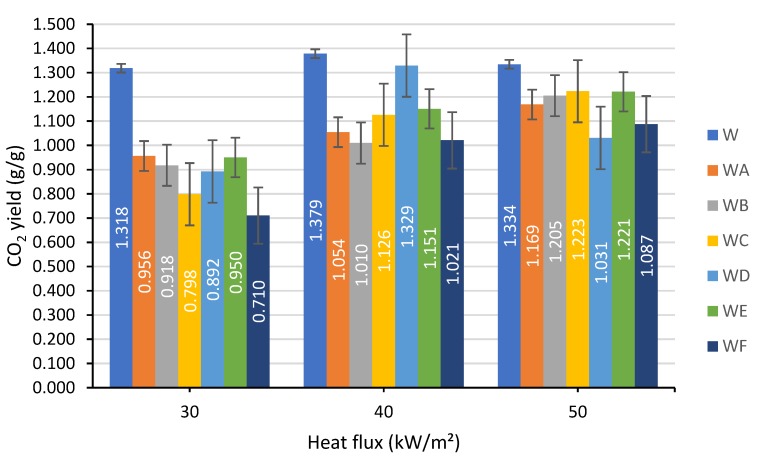
The CO_2_ yields measured at a heat flux of: 30, 40 and 50 kW/m².

The production trends for carbon monoxide CO and carbon dioxide CO_2_ are presented in Figure 7 and Figure 8.

The yield of CO_2_ released per gram of burning unprotected wood was not significantly affected by the intensity of thermal flux and remained in the region between 1.3 and 1.4 g/g. All the coated specimens were characterized by the lower CO_2_ production, with the formulation F performing better than the others. As for the toxic component of fire effluent, carbon monoxide, the application of the formulation F onto the wood resulted in a slight reduction of CO yield from 0.037 g/g to 0.029 g/g at 30 kW/m² heat flux and from 0.054 g/g to 0.041 g/g at 50 kW/m². On the other hand, at 40 kW/m², the production of CO was found to be higher for all the coated wood samples. The CO and CO_2_ production results were varied and dependent on the components used within the coatings and the thermal flux that they were exposed to.

#### 3.1.6. Smoke Production

**Figure 9:**
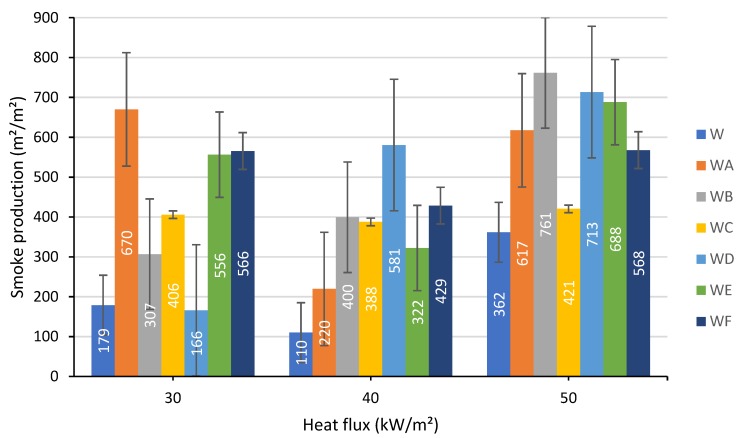
Smoke production measured at a heat flux of: 30, 40 and 50 kW/m².

Generally, the exposure of the wood samples coated with the intumescent formulations A–F at all three levels of heat flux contributed to a growth of the smoke production compared to the uncoated wood, with the exception of the coating D at 30 kW/m² when the smoke production slightly dropped (Figure 9).

#### 3.1.7. Back Surface Temperature

**Figure 10:**
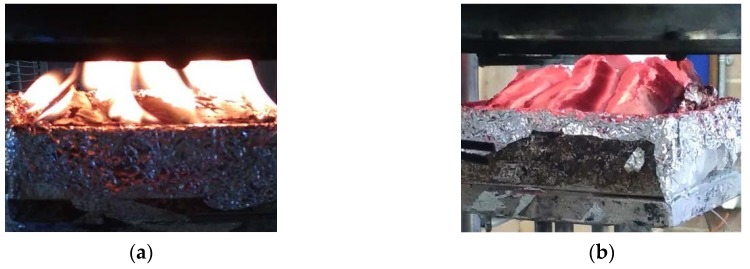
Wood samples coated with the formulation D after a 20 min exposure to a heat flux of: (**a**) 30 kW/m² and (**b**) 50 kW/m².

**Figure 11:**
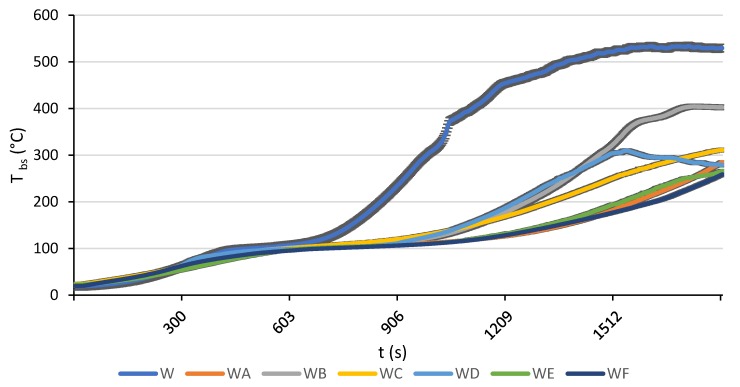
Back surface temperature versus time for unprotected and protected wood specimens exposed to a heat flux of 30 kW/m².

In this section, the back surface temperature (T_bs_) values measured at 30 kW/m² are reported. In the case of the exposures to 40 and 50 kW/m² heat fluxes, the temperature readings varied widely. It appeared that the wood sample tends to deform and bend under the influence of heat. This phenomenon seemed more pronounced when the heat flux is intensified. For instance, Figure 10 clearly demonstrates that for the same duration (20 min), a sample exposed to a lower heat flux (Figure 10a) had a flatter top surface than the one exposed to a higher heat flux (Figure 10b). This bending deformation created a gap between the back surface and a fitted thermocouple, which made the temperature measurements of the back surface less reliable. This effect was also observed at a lower flux, but it was developed more slowly. It was not possible to note exactly when the contact between the surface and the thermocouple was lost and how big the gap was. The bigger the gap, the lower the accuracy of the temperature measurements on the back surface of the wood substrate. Therefore, when analysing the temperature data shown in Figure 11, it must be kept in mind that the reliability decreases as we progress along the time axis.

The back surface temperature versus time curves for the uncoated and coated wood samples measured during cone calorimetry runs at 30 kW/m² heat flux is presented in Figure 11.

When a sample protected with the intumescent formulation was exposed to a heat flux of 30 kW/m² the coatings reacted accordingly, with the expected foam-forming and a production of a carbonaceous protective layer. From about 650 s onwards, the back-surface temperature of the uncoated wood sample increased rapidly and remained about 100–250 °C higher compared to the painted specimens. It is evident that the intumescent action of all tested coatings led to a marked delay corresponding to the start of the back surface temperature growth. In addition, the temperature rises significantly slower for all the protected specimens as opposed to the unprotected one. The decline of T_bs_ recorded at around 1500 s on the graph for the sample with the coating D can be attributed to a detachment of the substrate from the thermocouple (Figure 11). It can be concluded that the most effective thermal protection was offered by the coating F, combining two industrial and two bio-fillers (TiO_2_:Al(OH)_3_:RHA:ES = 2.5 wt.%:2.5 wt.%:2.5 wt.%:2.5 wt.%,), closely followed by the coating A and then by the coating E. Indeed, the increase in T_bs_ for the wood sample coated with the formulation F was delayed to 990 s with the highest temperature reaching around 250 °C, compared to 530 °C maximum for the uncoated sample.

### 3.2. Raman Spectroscopy Analysis

The burnt upper layer of the specimens obtained after the cone calorimetry runs at 40 kW/m² have been analysed by the Raman spectroscopy. The bands intensities and the ratios of interest are summarized in Table 2. The raw spectral data is provided as Supplementary Materials (Figure S1).

The D/G ratio greater than 1 indicated a predominantly disordered state of matter (a diamond-type char structure), while the ratio of less than 1 pointed out at predominantly ordered state of matter (a graphite char structure) in the carbonaceous protective layer [27,28]. The char residue obtained from the wood substrate protected with the coating A, containing titanium dioxide and RHA fillers, was characterized by the most ordered char structure, very similar to the one of unprotected wood, whilst the char from the sample coated with the formulation B, based on the industrial fillers only, was the most disordered. However, this is an initial result and before confirming it, more investigations on chars obtained at different heat fluxes are required. 

## 4. Conclusions

Cone calorimeter tests at varied levels of thermal flux ranging from 30 to 50 kW/m² were conducted to investigate the thermal performance of different waterborne intumescent coatings for fire protection of timber materials. The flammability of softwood was significantly reduced when a protective layer of the intumescent coatings, containing both industrial and bio-fillers, was applied onto its surface. This was gauged from the notable decreases in HRR, pHRR and EHC values and from the delay to the main peak of HRR registered for the protected specimens of wood. The main advantage of the tested intumescent coatings was found to be in the significant reduction of the back surface temperature, meaning that the level of burning and degradation through the entire depth of the sample is much lower compared to the unprotected sample. 

Although the yields of CO, CO_2_ and smoke production were measured in the cone calorimetry runs the results were found to be inconclusive. There is a need to carry out further characterizations of coated substrates, for example, by coupling the flammability apparatus with FT-IR or GC-MS instruments that would allow detection of other gaseous products of combustion.

In general, the coating F, which incorporated titanium dioxide, aluminium trihydroxide, eggshell and rice husk ash, provided better fire protection for wood compared to the other tested formulations. However, these are the initial findings and the formulations should be developed further, for example, by using only bio-fillers in their compositions. Also, several large-scale tests are strongly recommended to validate the obtained results.

Overall, the results of this study can have a significant impact on the fire classification of timber materials, as the incorporation of industrial and biological fillers, into the intumescent formulations leads to the improved fire resistance of timber and, potentially, to a reduced fire spread.

## Figures and Tables

**Table 1:** The amounts of industrial and bio- fillers incorporated into the intumescent coatings.

Coating	Type of Fillers			
	Industrial		Biological	
	TiO_2_ (wt.%)	Al(OH)_3_ (wt.%)	RHA (wt.%)	ES (wt.%)
A	5.00	-	5.00	-
B	5.00	5.00	-	-
C	5.00	-	-	5.00
D	3.40	3.30	3.30	-
E	3.40	3.30	-	3.30
F	2.50	2.50	2.50	2.50

**Table 2:** D and G bands intensities, and D/G ratios.

Specimen	D-band Intensity	G-band Intensity	D/G Ratio of Intensities
W	666.49	787.95	0.85
WA	465.93	548.93	0.85
WB	3524.93	3477.57	1.01
WC	8610.09	9000.80	0.96
WD	2491.69	2514.54	0.99
WE	8124.40	8552.50	0.95

## Supplementary Materials

The following is available online at https://www.mdpi.com/2073-4360/12/4/757/s1, Figure S1: The intensities of peaks from Raman spectra recorded on char residues collected post-cone calorimetry tests at 40 kW/m², for wood specimens coated the with formulations: A—(a), B—(b), C—(c), D—(d), E—(e), F—(f), or uncoated—(g).
