# Supplementary material for: Waterborne Intumescent Coatings Containing Industrial and Bio-Fillers for Fire Protection of Timber Materials
Source: Polymers (Basel). 2020 Mar 31;12(4):757. doi: 10.3390/polym12040757 (PMC7240737; doi:10.3390/polym12040757)
Supplement: Supplementary file 1 [file polymers-12-00757-s001.pdf]

polymers

MDPI

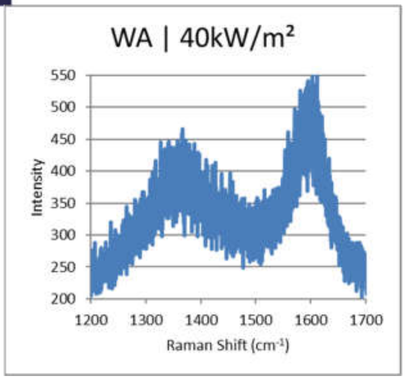

(a)

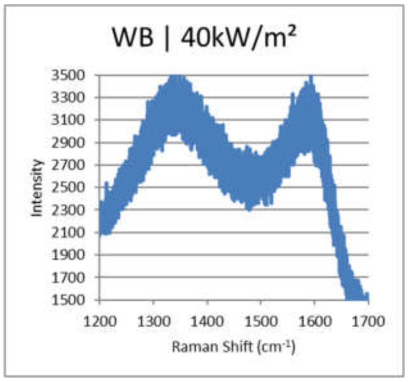

(b)

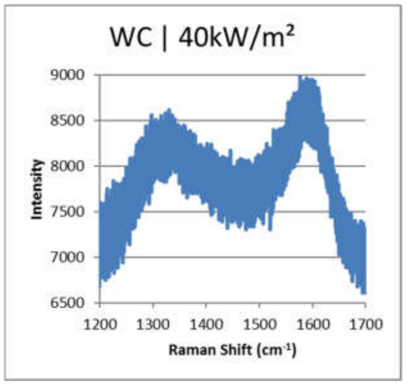

(c)

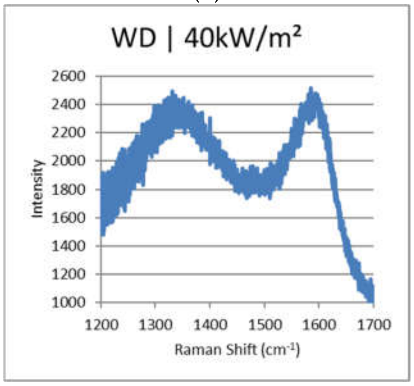

(d)

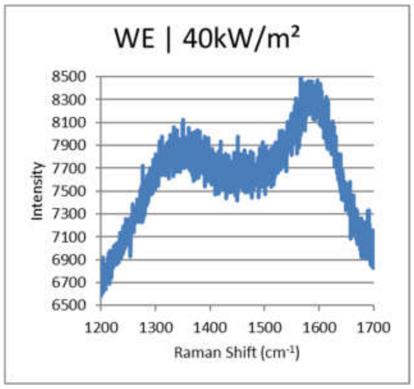

(e)

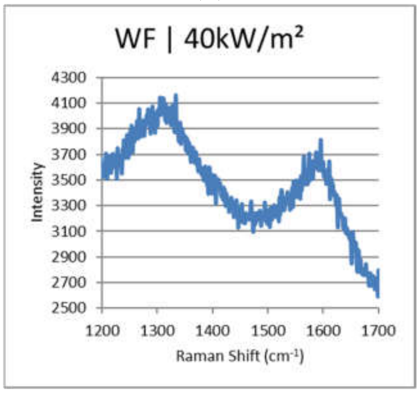

(f)

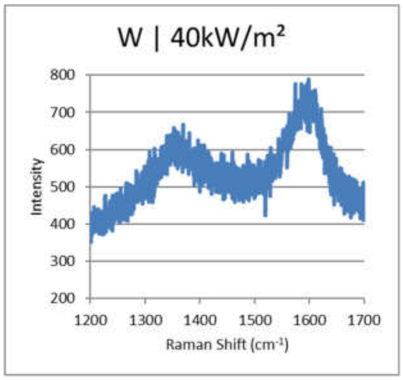

(g)

Figure S1. The intensities of peaks from Raman spectra recorded on char residues collected post-cone calorimetry tests at 40 kW/m^2, for wood specimens coated the with formulations: A - (a), B - (b), C - (c), D - (d), E - (e), F - (f), or uncoated - (g).
